# A Novel Virtual Reality Intervention Combining Movement Exercises and Body Illusions for the Treatment of Chronic Back Pain: Prospective Feasibility Study

**DOI:** 10.2196/81051

**Published:** 2026-03-30

**Authors:** Isabel Neumann, Stefan Lindner, Yevgeniya Nedilko, Ralitsa Zhivova, Michael Gödde, Tobias Tischer-Zeitz, Heike L Rittner, Ivo Käthner

**Affiliations:** 1 Department of Clinical Psychology and Psychotherapy, Institute of Psychology, University of Würzburg, Marcusstraße 9-11, Würzburg, Bavaria, 97070, Germany, +49 931-3189156; 2 Department of Anaesthesiology, Intensive Care, Emergency and Pain Medicine, Centre for Interdisciplinary Pain Medicine, University Hospital Würzburg, Würzburg, Bavaria, Germany; 3 Biomi Non-Profit UG (Ltd), Frankfurt, Germany; 4 videoreality GmbH, Frankfurt, Germany; 5 Department of Medicine, HMU Health and Medical University, Potsdam, Brandenburg, Germany; 6 Department of Physiological Psychology, University of Bamberg, Bamberg, Bavaria, Germany

**Keywords:** immersive virtual reality, chronic low back pain, gamification, kinesiophobia, feasibility

## Abstract

**Background:**

Virtual reality (VR) has proven effective in delivering nonpharmacological interventions to reduce acute and chronic pain. For the treatment of nonspecific chronic low back pain (CLBP), it offers benefits over traditional treatment options, such as the possibility of gamified movement exercises with real-time performance feedback and virtual embodiment. We implemented a novel immersive VR intervention (a serious game) that combined these elements.

**Objective:**

This study evaluated the feasibility, tolerability, and initial clinical efficacy of the gamified VR intervention.

**Methods:**

Patients with CLBP (n=20; mean age 47, SD 14 years; symptom duration >3 months to ≤5 years; convenience sample) took part in a prospective, single-arm, and preregistered trial over 9 weeks. The VR therapy phase lasted 3 weeks, and there were 2 VR sessions per week conducted at the University Hospital Wuerzburg (Germany). Before the therapy phase, there was a 2-week baseline phase, and the posttherapy phase lasted 4 weeks. During the sessions, patients wore a head-mounted display. In VR, they embodied a virtual avatar and performed gamified movement exercises. Participants were immersed in a virtual toy factory, and they had the task of teaching 5 different toys how to move. They received real-time feedback on performance through a hologram overlaying their avatar. Based on performance, movements to be performed became gradually more difficult (graded exposure). Primary outcome measures were adherence and side effects for assessing feasibility and tolerability (Simulator Sickness Questionnaire), and pain intensity ratings (numerical rating scale 0‐10) were used for assessing initial clinical efficacy. Secondary outcomes included back- and task-specific functioning and questionnaires to further test initial clinical efficacy and assess fear-avoidance beliefs.

**Results:**

Adherence was high (18/20, 90%). Participants indicated lower pain in the posttherapy phase compared with baseline levels (mean difference 0.73, CI 0.27-1.19; *t*_16_=3.38; *P*=.004; *d*=0.82). There were only few and minor side effects. Task- and back-specific functioning improved (ie, performing daily life activities; Back Performance Scale: *F*_2,34_=4.53; *P*=.02; η^2^_g_=0.04; Roland-Morris Disability Questionnaire: *F*_2,26_=4.73; *P*=.02; η^2^_g_=0.08), and movement restrictions decreased (*F*_2,32_=10.82; *P*<.001; η^2^_g_=0.06). There were no changes in the psychological outcome measures (eg, fear avoidance beliefs). Across all VR sessions, study participants reported high levels of fun (mean 8.07, SD 1.99).

**Conclusions:**

We implemented a gamified immersive VR intervention for the treatment of CLBP. The combination of gamified movement exercises and virtual body illusions is unique, and for the first time, it included real-time feedback via a hologram overlaying the virtual avatar of the user. The study demonstrated the feasibility and safety of the intervention. Initial tests of the clinical efficacy revealed positive effects on pain, physical functioning, and daily activities. However, these did not reach the thresholds of clinical importance. A randomized controlled trial is needed to test the specificity of the effects.

## Introduction

### Background

About 1 in 5 people experience chronic pain, with chronic back pain being the most common disorder [1,2]. Chronic pain can be a debilitating condition negatively affecting quality of life and mental health for the individual and leading to high societal costs [3-5]. Pharmacological treatments are often not effective and are associated with substantial side effects and risks, such as sedation, gonadal dysfunction, sleep disturbances, and abuse of opioid treatments [6]. Hence, there is a need for nonpharmacological treatments, especially as interdisciplinary multimodal pain therapy programs are considered the gold standard for day patients [7]. Virtual reality (VR) is a powerful technology to deliver nonpharmacological treatments for acute and chronic pain due to its immersive properties [8-10].

### Prior Work

Early VR studies focused on distraction and induction of positive affect for the treatment of acute pain [11-15]. More recent VR studies for chronic pain explored the feasibility of implementing a variety of treatments that go beyond these mechanisms, including pain education and pain relief skills training [16,17], embodiment [18-21], physical exercises [22-24], and mindfulness-based interventions [25]. In short, VR allows the implementation of traditional treatment approaches while employing additional, VR-specific mechanisms (refer to [9] for a review).

For nonspecific chronic low back pain (CLBP), pain-related fear and subsequent avoidance behavior are major factors for pain chronification and maintenance [26-28]. The fear-avoidance model of CLBP offers a cognitive-behavioral explanation for the development and maintenance of chronic pain based on approach and avoidance behaviors [27,28]. While protective behavior and movement avoidance can be useful for a limited period of time after an injury, perceiving pain as highly threatening can lead to catastrophizing, pain-related fear, and consequently avoidance through negative reinforcement. If maintained and generalized to novel situations, avoidance negatively affects valued activities and can lead to pain hypervigilance and depressive symptoms, which in turn negatively affect pain, resulting in a vicious circle. Accordingly, pain-related fear, catastrophizing, pain hypervigilance, and avoidance are predictors for chronic pain development (eg, [29-35]) and for experienced pain intensity and disability associated with pain [29,35-37], and they are associated with the status of pain recovery at a later time point [38,39]. By systematically reducing pain-related fear and avoidance behaviors, graded exposure in vivo can be a strategy to interrupt the vicious circle [40].

Physical exercise is a recommended first-line treatment for CLBP [41,42]. Systematic reviews suggest that treatment adherence is key to its efficacy in reducing pain and disability [43,44] but that adherence outside the clinic is low [45]. For CLBP, VR offers several advantages over traditional treatment approaches [46]. It can increase user motivation and engagement through gamification [23,47], offers the possibility of precise real-time feedback of movements, and holds the promise of standardized therapies that mitigate costs associated with supervision necessary during standard care. In addition, VR therapy has an immediate analgesic effect through distraction [48], which might help persons with kinesiophobia to exercise despite pain-catastrophizing thoughts and pain-related fear.

To reduce acute pain, previous VR interventions often immersed participants in pleasant virtual scenarios to heighten the mood and, thereby, achieved significant pain reduction [13-15]. For chronic pain and nonspecific CLBP in particular, regular exercise can be a key to recovery. Studies from the field of sports science investigated how affect during exercise influences future physical activity behavior [49]. In line with the hedonic theory, initial evidence suggests that positive affect during exercise increases the likelihood of future physical activity behavior [50] and negative affect reduces the likelihood [51] (see [49] for a review). VR appears to be a particularly suited method to induce a pleasurable exercise experience through high levels of embodiment, presence, and interactivity [52-54], and, thereby, promote future exercise. Gamified exercises could be a means to increase fun and could lead to higher adherence for patients with CLBP, as suggested by a previous article [46].

Furthermore, virtual embodiment could help to correct distorted body perceptions or maladaptive beliefs about the body (eg, fragile back) that are common in CLBP [55-58]. Although the benefits of VR for the treatment of CLBP are promising, there are very few interventions in immersive VR to date [10].

### Goal of This Study

For this study, we implemented a novel immersive VR therapy that combines gamified movement exercises designed as graded exposure therapy with innovative real-time feedback and virtual body illusions. Specifically, participants were immersed in a virtual toy factory presented through a head-mounted display (HMD). Within the virtual toy factory, study participants had the task of teaching 5 different toys how to move. Meanwhile, they received real-time feedback on performance through a hologram overlaying their avatar. Based on performance, movements to be performed became gradually more difficult (graded exposure). According to the guidelines for clinical VR studies in health care proposed by a previous article [59], our study was a VR phase 2 trial that needed to be conducted prior to a randomized controlled trial (VR phase 3). We focused the evaluation of the intervention on feasibility and tolerability, and gathered data to assess initial clinical efficacy, with a focus on pain reduction as the main clinical outcome.

## Methods

### Study Design

The feasibility, tolerability, and initial efficacy of a novel VR intervention for CLBP were tested in a prospective, single-arm, proof-of-concept study. The study protocol was approved by the Ethical Review Board of the Faculty of Medicine of the University of Würzburg (190/22-am) and preregistered at the German Clinical Trials Register (ID DRKS00031535). We adhered to the procedures described in our study registration. The study took place from May 2023 to February 2024.

### Ethical Considerations

Ethics approval to conduct this research was obtained from the Ethics Committee of the University of Würzburg prior to its start (IRB 190/22-am; November 2022). All study procedures were conducted in accordance with the Declaration of Helsinki. In particular, the study protocol, including the intervention and data collection methods, was considered to comply with the ethical standards for research involving human participants, and data were collected and stored in line with the European Union data protection law (General Data Protection Regulation [GDPR]). All participants provided written informed consent before participation. The consent form outlined the purpose, procedures, risks, benefits, and voluntary nature of participating in the study in plain terms. Participants were informed of their right to withdraw at any time without penalty. Research data were deidentified to ensure the privacy of all participants. Personal identifiers were not linked to the study data. No identifiable information will be shared with external parties or institutions. Electronic records were stored securely on password-protected devices. Participants provided consent that their anonymized data can be stored in a public repository to allow for secondary data analysis. Study participants received €60 (approximately US $70.75) for their participation.

### Participants

We recruited 20 study participants with CLBP via convenience sampling through internal postings at the University Hospital Würzburg, Germany, and newspaper advertisements. The ideal sample size was calculated a priori with G*Power (version 3.1.9.2 [60]) based on an expected medium to large effect size (*d*=0.77) for pain reduction, with a power of 0.8 and an α value of .05. This yielded 16 study participants, and to account for dropouts, we planned on recruiting 20 participants. The inclusion criteria were as follows: age 18‐65 years; diagnosis of nonspecific CLBP (symptom duration >3 months to ≤5 years and current pain intensity of ≥4/10 on a numerical rating scale [NRS]); and sufficient vision and language comprehension for the intervention. The exclusion criteria were as follows: specific back pain (eg, spinal canal stenosis, disc protrusion, and carcinoma); other chronic pain conditions (eg, fibromyalgia); current neurological or mental disorders; and other current diseases, including infectious, metabolic, endocrine, and severe internal secondary diseases. Participants were also excluded if they were receiving antidepressant medication, they were pregnant, or their glasses did not fit under the HMD. The inclusion and exclusion criteria were checked with structured questionnaires in case no current patient record was available. The mean age of the included participants was 46.85 (SD 14.25; range 24‐65) years, and the mean duration of back pain was 3.00 (SD 1.49; range 1‐5) years. Out of the 20 included participants, 13 were female.

### Outcome Measures

#### Primary Outcome Measures

##### Feasibility and Tolerability

To assess feasibility (primary outcome), we assessed adherence and side effects. We defined it as a success if ≥80% of participants completed the intervention, no serious side effects occurred (eg, injuries caused by falls), and ≤20% of participants experienced cybersickness (assessed with the Simulator Sickness Questionnaire [SSQ]) [61].

##### Pain Intensity

The co-primary outcome measure was pain intensity. The participants were asked to rate their pain intensity with the Patient-Reported Outcomes Measurement Information System 29 (PROMIS 29) scale from 0 (no pain) to 10 (worst pain imaginable) each day at a fixed time (when they usually experience high pain). We compared the mean ratings of the baseline period (baseline pain levels) prior to the intervention with the mean ratings assessed in the posttreatment phase (posttreatment pain levels).

### Secondary Outcome Measures

The secondary outcome measures described in the subsections below relate to the real-life impact on physiological outcomes (Range of Motion and Other Behavioral Measures subsection) and to the fear-avoidance model of chronic back pain that influenced the design of the intervention (Psychological Measures subsection). We investigated the initial impact on movement restrictions and daily life activities. The measures described below are a combination of subjective, patient-reported measures and objective tests.

#### Range of Motion and Other Behavioral Measures

As a secondary measure, we analyzed range of motion (ROM). A physiotherapist measured ROM in a standard physical assessment and classified ROM loss in 4 clinically important categories (nil, minimal, moderate, and major), as generally used in the McKenzie Method of Mechanical Diagnosis and Therapy (MDT) [62]. The reliability of the MDT has been accepted, and this method uses a practicable way to assess clinically important differences in ROM change [63]. The possible restrictions in ROM were assessed at the 3 clinical assessments (prestudy, poststudy, and follow-up). To quantify the classification of movement restrictions, we assigned a numerical value to every class (nil=0, minimal=1, moderate=2, and major=3). An overall score was calculated for each person and each time point for an objective comparison of ROM changes.

For measuring individual patient-related activities, we asked the participants to fill in the Patient-Specific Functional Scale (PSFS) [64]. In this questionnaire, the participants are required to state 3 to a maximum of 5 activities that they are unable to perform or only perform with difficulty due to back pain and that they would like to improve. They rate each of the named activities on a Likert scale from 0 (unable to perform the activity) to 10 (can perform the activity at the same level as before the injury or problem), with a higher score indicating an improvement in functionality. The minimal clinically important difference (MCID) for the mean value of the 3 rated activities is 2 points [65]. We administered the German version of the Roland-Morris Disability Questionnaire (RMDQ) [66,67] to measure how the participants’ back pain restricts specific activities. The participants were required to tick 1 (yes) or 0 (no) for restrictions in certain activities, resulting in a score ranging from 0 to 24. The minimal detectable change (MDC) is 2‐3 points [68]. At the end of the last session and at the end of the poststudy phase, we collected responses to the modified Patient Global Impression of Change (PGIC) [69]. The participants were asked to specify the changes related to their painful condition on a Likert scale from 1 (no change or the condition has gotten worse) to 7 (a great deal better and a considerable improvement that has made all the difference). Together with a physiotherapist, the participants also completed the Back Performance Scale (BPS) [70] for the evaluation and assessment of everyday functions of the lumbar spine on a Likert scale from 0 (no restriction) to 3 (massive restrictions). The BPS consists of 5 functional tests to measure mobility and load capacity in the most common sagittal plane of the spine (sock test, long-sitting test, finger-floor test, lift-up test, and lifting test), resulting in scores of 0‐15 points. The MCID is 3.6 points [71].

#### Psychological Measures

We assessed beliefs about the influence of physical activity and work on pain and psychological distress, and the extent to which pain is perceived as threatening. To this end, the participants were asked to complete the German version of the Fear Avoidance Beliefs Questionnaire (FABQ) [72,73] on a Likert scale from 0 (completely disagree) to 6 (completely agree). The score range is 0‐24 for the subscale physical activity and 0‐42 for the subscale work, with higher values indicating more fear-avoidance beliefs. Regarding chronic nonspecific low back pain, the MDC is 3.69 points for the subscale physical activity and 5.95 points for the subscale work [74]. The participants were also asked to fill in the PROMIS 29 [75,76] on a Likert scale from 1 (not at all) to 5 (a lot). The score range is 1‐5 for each of the 7 domains (physical functioning, anxiety, depression, fatigue, sleep disturbance, limitations due to pain, and participation in social roles and activities) to measure physical, mental, and social health. The MCID is between 3 and 5 points [77]. According to the fear-avoidance model, if individuals perceive pain as highly threatening, it can lead to catastrophizing. Therefore, we assessed pain catastrophizing via the German version of the Pain Catastrophizing Scale (PCS) [78,79] on a scale from 0 (not at all) to 4 (always). The score range is 0‐52, with an MDC of 7.73 points [80]. We used the German 11-item version of the Tampa Scale for Kinesiophobia (TSK) [64,65], which requires the participants to indicate their agreement with statements related to painful movement-related anxiety on a Likert scale from 1 (strongly disagree) to 4 (strongly agree). The score range is 11‐44, with higher scores indicating stronger kinesiophobia, and the MDC is 5.6 points [66].

### VR Interface and Hardware

During the VR intervention, the participants wore an HMD (Oculus Meta Quest 2) and held controllers (Oculus Meta Quest 2 tracking sensors) in both hands while standing. Their movements were tracked by an Azure Kinect DK system (Microsoft Corp) positioned in front of them.

The virtual environment was created with Unity (Unity Software Inc). For the intervention, we implemented gamified movement exercises, for which the participants were immersed in a virtual toy factory. Within the virtual toy factory, the participants had the task of teaching 5 different toys how to move.

We implemented movement exercises that were selected by a physiotherapist to be compatible with the goals and constraints of the planned VR intervention for the treatment of CLBP. It is unclear which exercise therapy is most effective for CLBP [81,82]. However, clinical practice guidelines on CLBP strongly recommend exercise therapy [42]. General activity of the participants appears to be more important than the type of exercise [83], and correctly performed exercise therapy is safe [84]. We selected standing movements in one place for safety reasons, as the real world is hidden from view, and so that the participants can always perceive the feedback provided by the hologram. The following movements, which are often restricted in patients with CLBP [65,70], were selected and performed repetitively: (1) lateral flexion of the lower spine (participants held their hips with their hands and moved the hips to the left and right sides), (2) squat (with a slightly wider stance and movement of the arms in flexion while squatting deeper for balance control), and (3) trunk rotation (in 2 different styles: one involving global rotation with arm movements like an archer, and the other with the hands at the hip and turning with the shoulder line) [62]. Prior to each movement exercise, the participants received detailed instructions in VR via a virtual screen. During the movement exercises, the participants saw a virtual hologram overlaying their virtual body and were asked to follow the movements of the hologram.

To determine the movement range to be performed, we conducted a usability study with 20 healthy participants prior to the clinical feasibility study. The mean value of the maximum ROM of the second to fourth best subjects was determined as the highest level of difficulty (level 3) for the participants with CLBP. We omitted the best value due to potential measurement errors or statistical outliers. The other 2 levels were calculated in equal parts from the difference between the mean threshold and the starting position (Multimedia Appendix 1).

**Figure 1.**
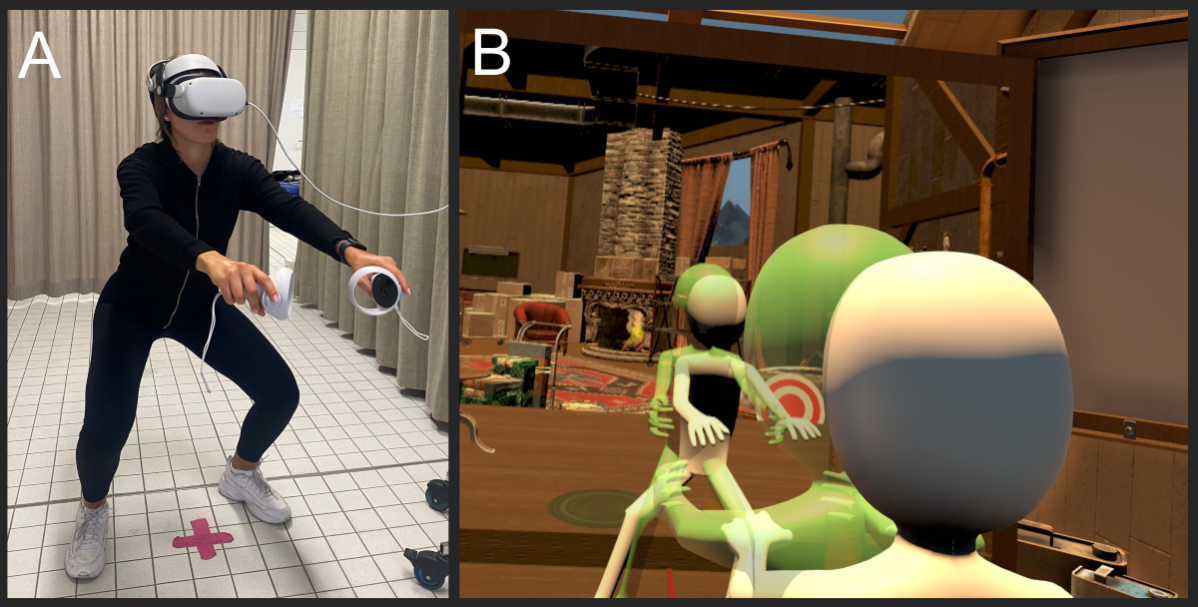
Illustration of movement exercises in virtual reality (VR). (A) A female person in VR equipped with a head-mounted display and controllers, who is executing a movement exercise. (B) View of the person in VR seeing her embodied body from a third-person perspective and a hologram for feedback of the movement exercise. Consent has been obtained from the depicted person for the publication of this image.

The hologram provided the participants with real-time visual feedback on their movement accuracy (Figure 1). In addition, they received a performance score and auditory feedback (eg, “well done” after they finished an exercise). The movement performance score was based on an overlap with the ideal movement pattern determined in the study with healthy participants. While the participants performed the movement, the hologram overlayed their virtual body (based on the ideal movement pattern). The score was based on a match (within a margin of error) between the movement performed (depicted by the avatar of the user) and the “ideal” movement (displayed through the hologram). A higher score was given if there was a closer match between both movements. Each movement exercise consisted of 3 difficulty levels that could be selected (ROM gradually increased with each level). An algorithm was used to increase the level of difficulty. All participants started at the first level for all exercises, and in each session, the participants performed all 4 movements at least once. If the pain intensity increased by more than 1 point on the NRS, the difficulty level of that exercise was not increased. The participants were allowed to try 2 of the other exercises at an increased level in the same session. In each subsequent session, the participants started at the previously performed difficulty level. The aim was to achieve the greatest ROM within the VR sessions without provoking a clinically relevant increase in pain.

### Embodiment of the Virtual Character

At the beginning of the VR session, upon entering the toy factory (after the calibration phase and pain ratings), the participants were told to accustom themselves with the virtual body. They saw the virtual body from a first-person perspective and were asked to perform movements. The virtual body moved according to their real movements that were tracked via the handheld controllers and the Azure Kinect sensor. This procedure was previously demonstrated to induce a full-body illusion (eg, [85,86]; see Multimedia Appendix 1). A virtual mirror was placed in front of the avatar for the participant to observe the movements of the body. In the second step, a virtual teleportation procedure was displayed (visual effect), and the avatar’s position was moved such that subsequently the participant observed it from a third-person perspective while keeping full agency over it.

### Procedure

**Figure 2.**
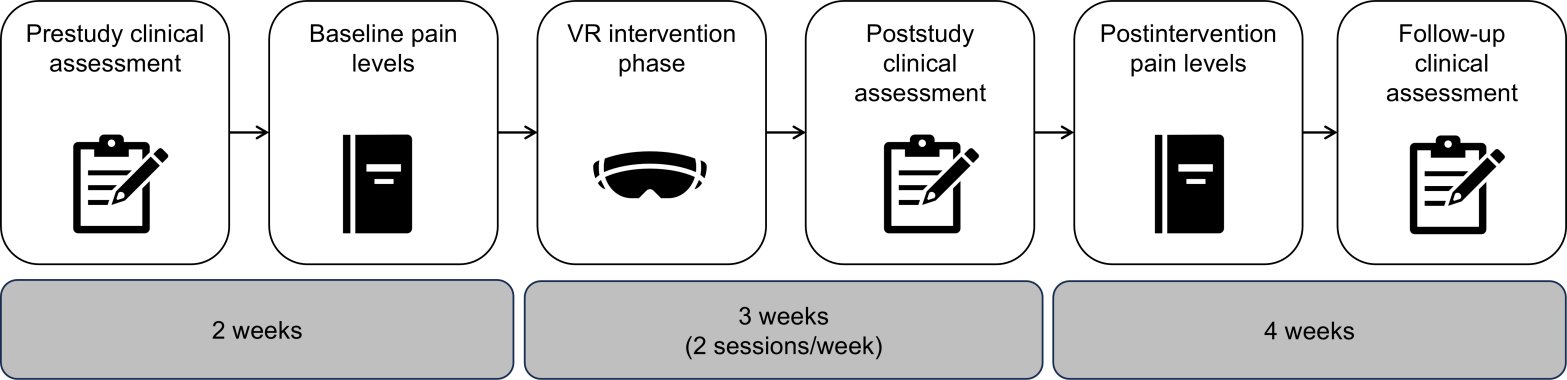
During the preintervention phase (2 weeks), study participants with chronic low back pain (n=20) filled in questionnaires, range of motion (ROM) was assessed, and baseline pain levels were assessed via a pain diary. In the intervention phase (3 weeks), the virtual reality (VR) intervention was conducted, participants filled in questionnaires, and ROM was assessed. In the postintervention phase (4 weeks), postintervention pain levels were assessed via a pain diary, participants filled in questionnaires, and ROM was assessed.

The prospective feasibility study lasted 9 weeks for each patient (Figure 2). Prior to the prestudy clinical assessment, the participants were screened according to the inclusion and exclusion criteria via a phone call.

In the preintervention phase, the participants underwent a medical examination, including an assessment of their ROM and a query of current medications (after providing informed consent). If eligibility was confirmed, the participants were asked to fill in the questionnaires that served as secondary outcome measures. In the following 2 weeks, the participants were asked to fill in a pain diary at the time of the expected highest pain intensity. They were also asked to estimate the maximum pain intensity at an individual time point at the end of each day on an NRS (ranging from 0 to 10). Furthermore, they were asked to indicate the duration and intensity of pain exacerbations and the possible use of pain medication.

**Figure 3.**
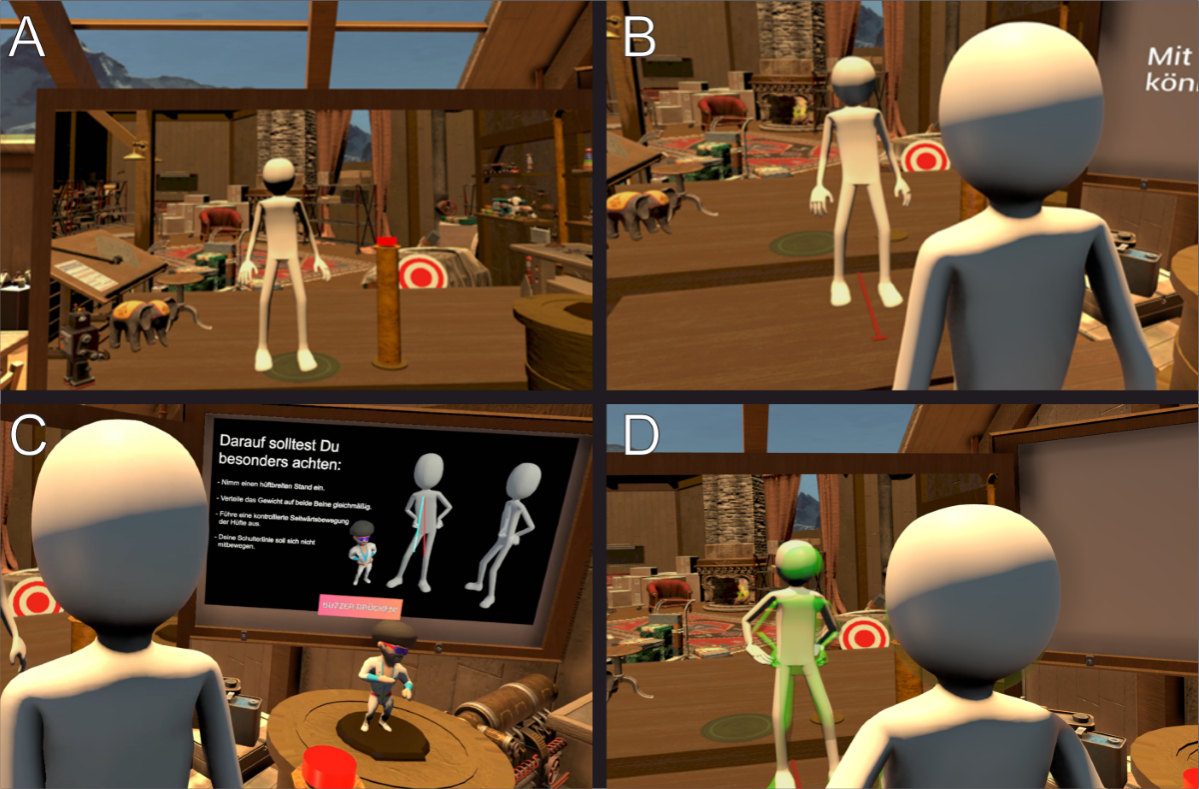
Overview of the virtual reality session procedure. (A) Upon entering the virtual toy factory, participants observed their avatar from a first-person perspective. Their movements were tracked, and the virtual body moved accordingly. They were standing in front of a virtual mirror, enabling them to see their whole body. (B) In the second step, participants observed their avatar from a third-person perspective. (C) Prior to each exercise, the required movements were demonstrated on a virtual screen. In the depicted case, lateral flexion of the lower spine was required to teach the toy how to dance (study participants held their hips with their hands and moved the hips to the left and right sides). (D) While participants performed the movements, they received real-time feedback through a hologram overlaying their avatar.

The intervention phase lasted 3 weeks and included 2 VR sessions per week (on separate days with at least 1 day off between sessions) with a duration of 30 minutes per session (Figure 3). The number of sessions per week was chosen to ensure regular exercise and make study participation manageable in daily life. The exercise duration was similar to the duration of a regular physiotherapy session. In each session, there were 6 exercises, and the participants practiced each exercise. The order of exercises could be chosen by the participants. The difficulty level of the movement exercises was gradually increased over the course of treatment. At the beginning of each VR session and after each movement exercise, the participants were asked to rate their current pain intensity. Furthermore, after each movement exercise (while being immersed in VR), the participants received feedback from the VR application about their performance. At the end of each VR session, the participants were asked to indicate their levels of presence (how much they felt as if they were in the virtual environment) and fun during the application on an NRS (ranging from 0 to 10).

After the last VR session, the participants were physiotherapeutically assessed again. ROM was measured, and they were asked to fill in the questionnaires that served as secondary outcome measures. Furthermore, they received instructions and information about movement exercises to be practiced at home at their own discretion during the poststudy phase. For this, they were sent 3 videos by email so that they could practice each exercise at least once a day. These 3 exercises (squat, lateral flexion, and rotation) in the standing position were the same as executed in VR and with the same number of repetitions.

In the postintervention phase, the pain diary was continuously filled in daily. The participants were asked to state in a retrospective verbal question how often they had practiced the movement exercises, to which all participants responded in the affirmative. A follow-up assessment took place 4 weeks after the last VR session, in which ROM was assessed and the secondary outcome questionnaires were filled in.

### Statistical Analysis

Primary outcomes in this study were pain intensity ratings, adherence, and side effects. Secondary outcomes in this study were changes in ROM, TSK, FABQ, PGIC, PSFS, BPS, RMDQ, PROMIS 29, and PCS scores from the prestudy clinical assessment to the poststudy clinical assessment.

The primary outcome measures were analyzed via frequencies (adherence and side effects) and 2-sided *t* tests (pain ratings). All secondary outcome measures were analyzed separately with repeated measures ANOVA (rmANOVA) with the within-subjects factor time (prestudy, poststudy, and follow-up). In case of significant main effects, 2-sided post hoc *t* tests were conducted. Regarding missing data, we used the Little MCAR (missing completely at random) test; however, the findings did not reach significance (all *P*>.05; see Table S1 in Multimedia Appendix 2 for the results of the Little MCAR test and the number of missing data per outcome variable). We have reported means and SDs. The significance level was set at *P*<.05. Statistical analyses were conducted in R 4.1.0 [87]. The rstatix package [88] was used for the rmANOVA, the lsr package [89] was used for *t* tests, the psych package was used for descriptives [90], and the ggplot2 package [91] was used for data visualization.

## Results

### Primary Outcome Measures: Feasibility, Tolerability, and Pain Relief

**Figure 4.**
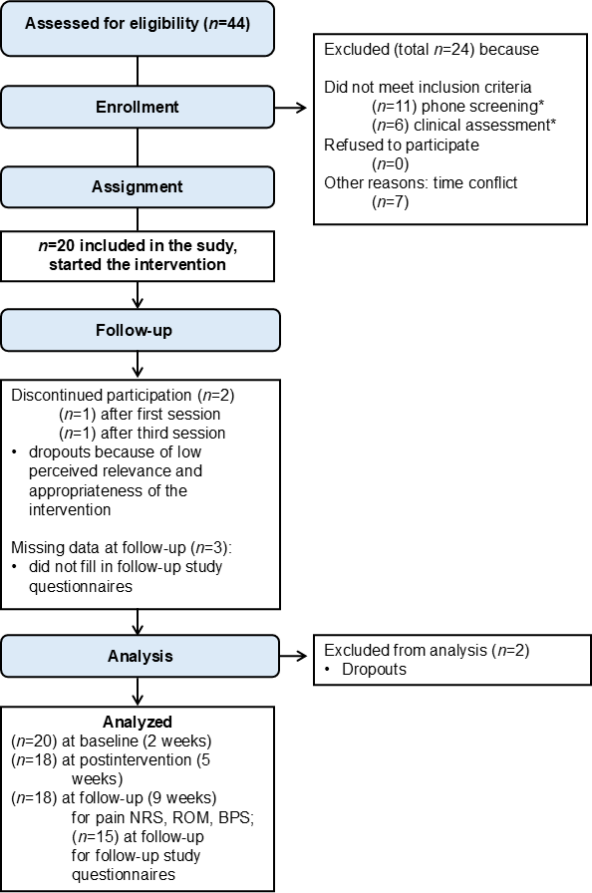
Participant flow diagram. Interested participants with chronic low back pain were screened via a phone call based on the inclusion criteria. They were then invited to a prestudy clinical assessment, and if they met the inclusion criteria, they were included in the study. All included participants experienced the virtual reality intervention in the intervention phase. BPS: Back Performance Scale; NRS: numerical rating scale; ROM: range of motion. *Phone screening: experiencing chronic back pain for >5 years (n=7), experiencing other pain (eg, thrombosis and chronic bone pain; n=2), being too old (n=1), and taking opioids (n=1); Clinical assessment: experiencing chronic back pain for >5 years (n=2), experiencing specific back pain (n=2), and localization of pain not fitting (thoracic and cervical spine; n=2).

The participant flowchart is presented in Figure 4. Out of the 20 study participants included, 18 (90%) completed the study, and no serious side effects occurred. Two study participants decided to stop participating (one participant after the first session, and the other participant after the third session in VR). One participant indicated not having fun and did not see it as relevant to an individual’s pain symptoms, and another participant perceived the toy factory scenario as not serious enough for treating the pain condition.

Regarding potential symptoms of cybersickness, there were only few and minor side effects assessed with the SSQ, with the most frequent relating to sweating, strained eyes, and difficulties in focusing with the HMD (see Table S1 in Multimedia Appendix 1 for the number of participants indicating SSQ symptoms per session and Figure S1 in Multimedia Appendix 1 for mean SSQ scores per session).

**Figure 5.**
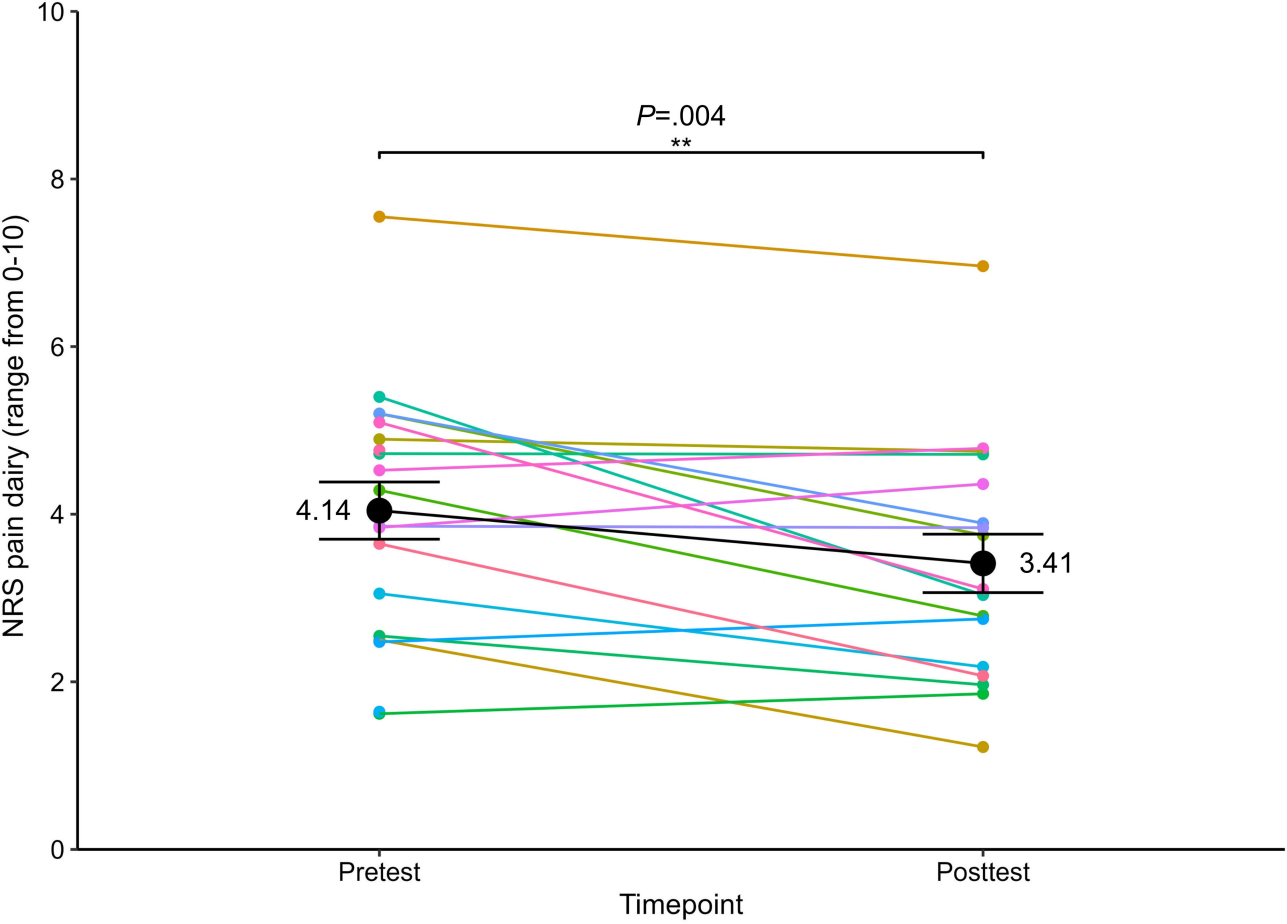
Pain relief from preintervention to postintervention. Means and SEs of baseline and posttreatment pain (black dots and line, measured via a numerical rating scale [NRS; 0‐10]), and means of individual study participants (colored dots and lines; n=18; 2-sided *t* test). *P≤.01.

We identified a significant pain reduction from baseline pain levels (mean 4.14, SD 1.44) to postintervention pain levels (mean 3.41, SD 1.44), with a mean difference of 0.73 (CI 0.27-1.19; *t*_16_=3.38; *P*=.004; *d*=0.82) (Figure 5). Baseline pain levels did not differ from day 1 to day 14 (*t*_14_=0.23; *P*=.82; *d*=0.06), demonstrating baseline stability. Among the participants who experienced pain reduction (n=13), 6 experienced pain reduction of ≥1 point and 1 experienced pain reduction of ≥2 points on the NRS.

There was no change in pain from immediately before the session (mean 3.94, SD 1.06) to after the session (mean 4.23, SD 1.32), with a mean difference of 0.30 (CI −0.90 to 0.30; *t*_17_=−1.04; *P*=.31; *d*=0.15) (see Figure S2 in Multimedia Appendix 1 for pain ratings before and after each VR session).

### Secondary Outcome Measures: ROM and Behavioral and Psychological Variables

**Figure 6.**
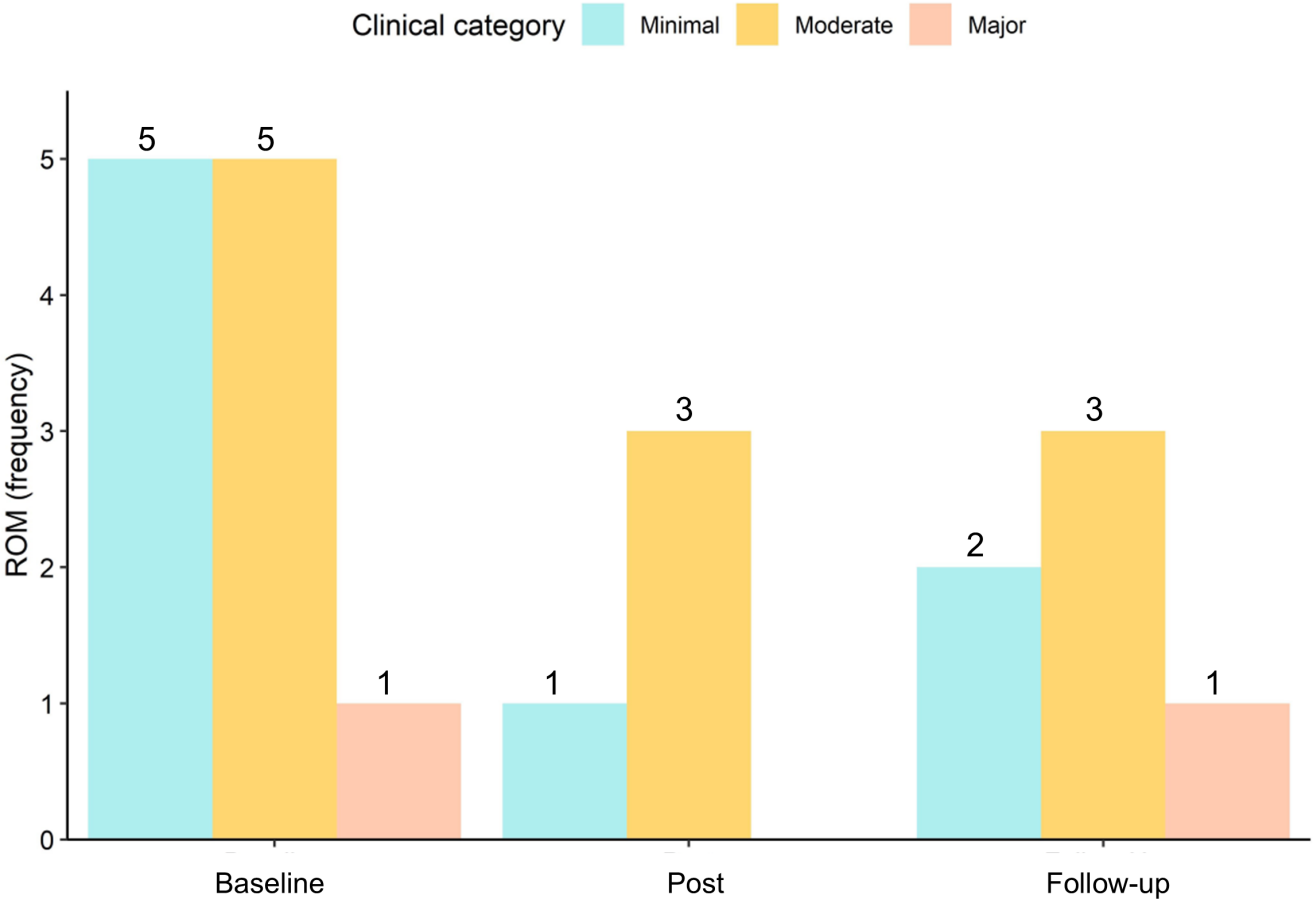
Reduced frequency of movement restrictions from preintervention (baseline) to postintervention and follow-up. ROM: range of motion.

There was a significant main effect of time on movement restrictions (*F*_2,32_=10.82; *P*<.001; η^2^_g_=0.06). Movement restrictions reduced from preintervention (sum score of 55) to postintervention (sum score of 22) (*t*_17_=3.70; *P*<.001; CI 0.45-1.66) and from preintervention to the follow-up assessment (sum score of 22) (*t*_17_=3.98; *P*<.001; *d*=0.94; CI 0.83-2.72). There were no significant differences from postintervention to the follow-up assessment (*t*_17_=1.79; *P*=.09; *d*=0.42; CI −0.13 to 1.57) (Figure 6).

**Figure 7.**
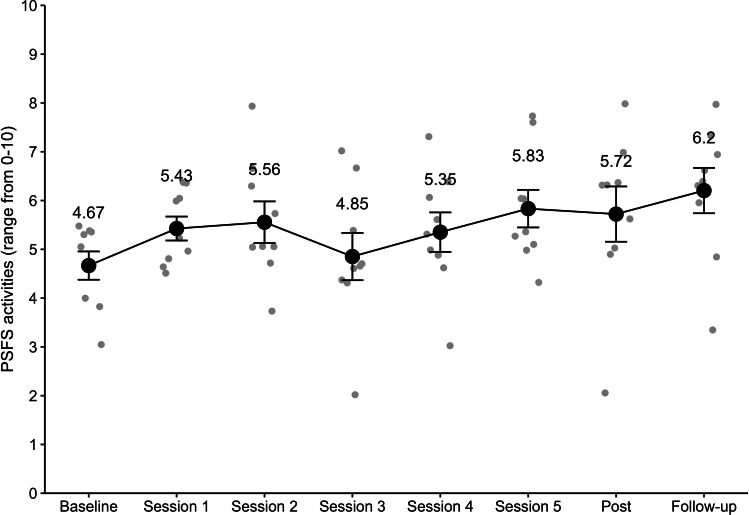
Improvement of function. Means and SEs (black) and individual scores (gray) over all 3 Patient-Specific Functional Scale (PSFS) activities in each session. Note that higher scores indicate a higher level of functioning.

Regarding the questionnaires addressing behavioral variables, for the BPS, there was a significant main effect of time (*F*_2,34_=4.53; *P*=.02; η^2^_g_=0.04). BPS scores reduced from preintervention (mean 2.06, SD 2.15) to postintervention (mean 1.56, SD 1.98) (*t*_17_=2.15; *P*=.046; *d*=0.51; CI 0.01-0.99) and from preintervention to the follow-up assessment (mean 1.17, SD 1.62) (*t*_17_=2.56; *P*=.02; *d*=0.61; CI 0.17-1.61). There were no significant differences from postintervention to the follow-up assessment *(t*_17_=1.28; *P*=.29; *d*=0.30; CI −0.25 to 1.03). For the RMDQ, a significant main effect of time was noted (*F*_2,26_=4.73; *P*=.02; η^2^_g_=0.08). RMDQ scores decreased from preintervention (mean 5.0, SD 3.11) to the follow-up assessment (mean 3.57, SD 3.01) (*t*_13_=2.59; *P*=.02; *d*=0.69; CI 0.24-2.62) and from postintervention (mean 5.86, SD 3.61) to the follow-up assessment (*t*_13_=2.43; *P*=.03; *d*=0.65; CI 0.25-4.32). There were no significant differences from preintervention to postintervention (*t*_13_=−1.21; *P*=.25; *d*=0.32; CI −2.39 to 0.68). Furthermore, there was a trend toward significance for the PSFS (*F*_2,16_=2.68; *P*=.10; η^2^_g_=0.21) (see Figure 7 for courses of functional improvement and Table S2 in Multimedia Appendix 1 for the frequency and improvements of the most common activities listed). Lifting, carrying, walking, and standing (Table S2 in Multimedia Appendix 1) showed clinically significant improvements from preintervention to the follow-up assessment (≥2 points) [65].

All other questionnaires (behavioral variables: PGIC; psychological variables: FABQ, PROMIS 29, PCS, and TSK) did not yield significant differences (all *P*≥.79). The results of all questionnaires are presented in Table S3 in Multimedia Appendix 1.

### Positive Evaluation of VR Application Measures

**Figure 8.**
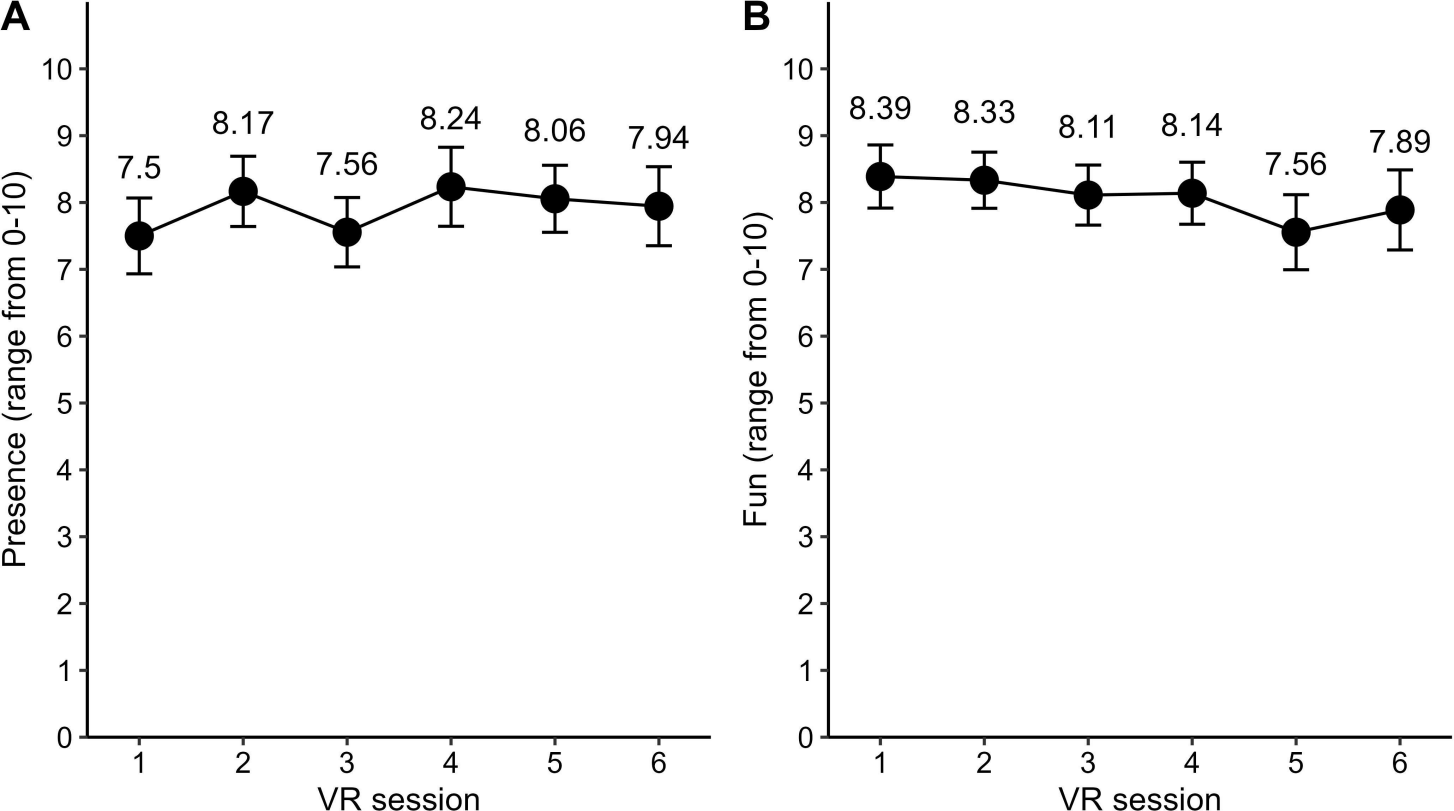
Levels of presence and fun. Means and SEs of the reported feeling of presence (A) and level of fun (B) in each session (both measured via a numerical rating scale [NRS; 0‐10]). VR: virtual reality.

Across all VR sessions, the participants reported high feelings of presence (mean 7.88, SD 2.01) and high levels of fun (mean 8.07, SD 1.99) (Figure 8).

## Discussion

### Principal Results

Our novel immersive VR intervention for chronic back pain follows an innovative approach in which users embody a virtual avatar, perform gamified movement exercises, and receive real-time feedback about their performance. We demonstrated the feasibility and tolerability of the intervention as 90% of participants completed the 3-week intervention phase, and there were no adverse events posing a danger to the health of the participants or indicating cybersickness. Importantly, as an initial test of the efficacy of the intervention, we focused on pain as the main outcome and assessed physical functioning and the influence on psychological variables in relation to the fear-avoidance model of chronic pain. In terms of effect size, the study revealed a large and statistically significant pain reduction from the 2-week baseline phase to the 4-week poststudy phase. However, this does not constitute a clinically meaningful reduction in pain over the treatment interval [92-94]. Notably, the intervention improved functioning (ie, performing daily life activities [PSFS and BPS]) and decreased movement restrictions, as demonstrated in ROM and RMDQ scores. However, the mean changes were below commonly cited thresholds for clinical importance. There were no effects on the scales assessing psychological outcomes. The results are promising, but a randomized controlled trial is needed to assess the specificity of the effects. The participants generally liked the intervention, as evident from the high fun rating in each session. Only 1 participant did not like the gamified task and dropped out after session 2. While gamified VR is suitable for the majority of participants, it might not be suited for everyone, as evident from the dropout of another study participant who had the impression that the intervention was not serious enough.

### Comparison With Prior Work

There are a few studies investigating the effects of interventions delivered via immersive VR to treat CLBP [10,95,96]. Our study extends the findings of a meta-analysis that showed positive effects of VR on back pain for non- and moderate-immersive virtual environments [10]. The only fully immersive VR study included in the meta-analysis revealed detrimental effects on pain intensity [97]. However, that study included mindfulness and cognitive behavioral therapy–based content, whereas our study investigated the effect of exercise. Using a subgroup analysis, the meta-analysis by Bordeleau et al [10] showed that the association between VR interventions and back pain intensity remained statistically significant only for VR exercises. While exercises are an effective and recommended first-line treatment for CLBP [41,42,98,99], adherence outside the clinic is often low [45]. It was previously demonstrated that gamified tasks in VR increase motivation [47]. Moreover, VR appears to be a particularly suited method to induce a fun experience through gamified tasks and high levels of embodiment, presence, and interactivity [46,52-54], and thereby, exercise in VR could serve as a gateway for future exercise [49]. In this study, we demonstrated the feasibility and tolerability of a gamified intervention delivered via VR, including performance feedback and virtual embodiment. We tested only the initial efficacy of the intervention, and when comparing the effects to previously published studies, it is important to keep the relatively low number of exercise sessions in mind (6 sessions).

Study participants with CLBP rated their pain daily before the intervention for 2 weeks and daily after the intervention for 4 weeks [97,100]. Only considering the same number of sessions as in our study, the average reduction in pain intensity was in a similar range in previous VR interventions [17,100,101]. Although the effect in our study was large in terms of effect size, it did not constitute a clinically meaningful effect in terms of an absolute pain reduction for most of the study participants [92]. This is likely due to several reasons. First, the mean pain ratings of the study participants during the baseline period were only slightly above the threshold for inclusion (≥4), indicating low to medium pain levels. Previous studies and a current meta-analysis demonstrated larger effects for higher baseline pain levels [8,17,102]. Second, the duration of the intervention was relatively short. Bordeleau et al [10] demonstrated the beneficial effects of VR when more than 12 sessions were performed. A recent study with persons with CLBP who took part in a behavioral skills–based VR program demonstrated a clinically meaningful pain reduction during home-based training [17]. That study was substantially longer than our study (56 daily sessions over the course of 8 weeks) and included different elements (pain education, relaxation/interoception, mindful escape via 360° videos, interactive games for pain distraction, and dynamic breathing).

In addition to preintervention and postintervention pain ratings (baseline vs follow-up phase), pain ratings before and after each VR session (assessed in VR) served to evaluate the immediate effects of the intervention and to document possible pain exacerbation caused by movements. We found no immediate changes in pain ratings in response to the intervention. Further, none of the participants ended the intervention due to pain induced by the required movements. This finding is in line with a usability study that gamified a lumbar flexion task in VR for persons with CLBP [24]. This demonstrates that the VR games distracted from pain otherwise caused by movements and encouraged participants to perform movements in VR that might be avoided in the real world due to fear of pain.

Partially based on the fear-avoidance model [27,28], our intervention was designed as a graded exposure therapy in VR, that is, ROM for the individual movement exercises was gradually increased (3 difficulty levels per exercise). In line with the results by Bordeleau et al [10], the VR intervention yielded measurable improvements in back and task-specific functioning, for example, in daily activities that were difficult or impossible to perform due to back pain, as measured in the PSFS. Participants in this study indicated functional impairments in typical activities [65] like walking, standing, bending, or carrying. Notably, lifting, carrying, walking, and standing showed clinically significant improvements [65], and there were gradual improvements over sessions. Functional tests of the lumbar spine (BPS) were conducted and revealed significant improvements over the course of the study. With the RMDQ, back pain was evaluated in the context of specific activities, and pain reductions over the course of the study were identified. Considering the low initial degree of disability, a difference of 1 point can be considered as an improvement [68]. The fact that the beneficial effects of functional measurements were not revealed immediately after the intervention but at follow-up can probably be attributed to persons with CLBP continuing to exercise after the intervention (as encouraged). This suggests that the VR intervention might serve as a gateway to continued beneficial exercises after the intervention. However, owing to the lack of a control group, the specificity of the results remains to be demonstrated.

While the intervention was also designed to have beneficial effects on kinesiophobia and fear-avoidance beliefs, effects on these outcomes, as well as on pain catastrophizing, could not be revealed due to the lack of clinically meaningful scores of the study participants at the beginning of the study. Specifically, all study participants with CLBP had low scores on the TSK [103]. Likewise, none of the participants had scores above 30 on the PCS, which would indicate a clinically significant level of pain catastrophizing [104]. For fear-avoidance beliefs assessed with the FABQ, none of the participants had scores above values for fear-avoidance beliefs related to work that are considered high, and for the physical activity subscale, only 1 participant had a high score (>15) that dropped to 3 (postintervention evaluation) [105,106]. However, another study discussed that, besides psychological variables such as catastrophic thinking, physiological adaptations related to the avoidance of certain movements could also play a crucial role in the maintenance of pain, emphasizing the role of treating functional impairments [107,108].

Overall, it is encouraging that there were only few and minor symptoms assessed with the SSQ, with most relating to sweating (probably due to exercising, especially in summertime, and wearing an HMD), followed by eye strain and difficulty focusing with the HMD. Vertigo was not present after any of the sessions, and stomach awareness and nausea were only reported by a single participant after 1 session. Therefore, the intervention is not likely to induce symptoms of cybersickness.

### Limitations and Outlook

Most importantly, we demonstrated the feasibility and tolerability of the novel intervention, and tests of the initial clinical efficacy are promising. However, it is important to stress that we conducted a single-arm study, and to demonstrate the specificity of the effects, a controlled study is necessary. The generalizability of the results is further limited due to the rather small sample size. The revealed effects did not exceed previously defined thresholds of clinical efficacy. The duration of the intervention was shorter compared to conventional physiotherapy for CLBP [109]. Hence, a longer duration of the intervention is desirable to increase its effects on clinical outcomes. The intervention is partially based on the fear-avoidance model of chronic pain, and while effective in reducing pain and behavioral outcomes, effects on cognition could not be revealed due to low scores on catastrophizing, fear-avoidance beliefs, and kinesiophobia of the study participants; hence, a larger or more selective sample is needed to shed further light on the potential of the intervention to affect these outcomes [107].

A fully immersive VR system has several advantages in terms of delivering a gamified environment, and higher levels of immersion have positive effects on pain through increased distraction [9,14,46]. Future studies could also explore the potential of VR to not only change the visual appearance of the body of the participants (as done in our study) but also manipulate the visual feedback in relation to movements, and investigate how overstated or understated movements displayed (compared to actual performed movements) affect exercise performance and effectiveness. In a previous study, an overstated or understated neck rotation in relation to neck pain was investigated [110]. Further, using a virtual cycling exercise, studies investigated how a match or mismatch between actual pedaling resistance and the virtual environment (higher/lower hill gradient) affects exercise performance [111] and perception of exercise effort [112]. Despite its benefits, immersive VR is also associated with a higher risk of collision with objects in the real environment because the HMD blocks visual input from the real environment. Given technological advances, augmented reality could be a promising alternative that utilizes the benefits of VR, such as gamification, while ensuring safe use (eg, showing obstacles in the real world while performing exercises) [113]. The present camera-based body tracking system allowed unrestricted movements of the participants and provided adequate accuracy for real-time feedback with the hologram. In order for it to work, a controlled environment with adequate lighting and high-contrast clothes were needed to properly detect the body. For a home environment or if more precise tracking is desired, tracking based on sensors attached to the body might be needed. Sensors attached to the body might also be preferable if biofeedback analysis is the relevant outcome measurement, as implemented previously [23].

### ttle Conclusions

We demonstrated the feasibility and safety of a novel VR intervention for nonspecific CLBP. It involved embodiment and gamified movement exercises in immersive VR with real-time feedback about performance provided through a hologram overlaying the avatar of the user. Almost all participants liked the intervention (high fun ratings), which is important as it can motivate patients to exercise and potentially increase exercise adherence in the long term. Despite its short duration of 6 sessions over the course of 3 weeks, positive effects on pain, physical functioning, and daily activities were noted. However, these effects fall short of reaching previously defined thresholds of clinical importance, and the study design does not allow to conclude on the specificity of these effects. Hence, a longer intervention is desirable, and a controlled study is needed to test its clinical effects.

## Supplementary material

10.2196/81051 Multimedia Appendix 1 Additional material to support the study.

10.2196/81051 Multimedia Appendix 2 Results of the Little missing completely at random test for each outcome variable.

## Abbreviations

BPS: Back Performance Scale
CLBP: chronic low back pain
FABQ: Fear Avoidance Beliefs Questionnaire
HMD: head-mounted display
MCAR: missing completely at random
MCID: minimal clinically important difference
MDC: minimal detectable change
MDT: McKenzie Method of Mechanical Diagnosis and Therapy
NRS: numerical rating scale
PCS: Pain Catastrophizing Scale
PGIC: Patient Global Impression of Change
PROMIS 29: Patient-Reported Outcomes Measurement Information System 29
PSFS: Patient-Specific Functional Scale
rmANOVA: repeated measures ANOVA
RMDQ: Roland-Morris Disability Questionnaire
ROM: range of motion
SSQ: Simulator Sickness Questionnaire
TSK: Tampa Scale for Kinesiophobia
VR: virtual reality
