# Supplementary material for: A Novel Virtual Reality Intervention Combining Movement Exercises and Body Illusions for the Treatment of Chronic Back Pain: Prospective Feasibility Study
Source: JMIR Serious Games. 2026 Mar 30;14:e81051. doi: 10.2196/81051 (PMC13035036; doi:10.2196/81051)
Supplement: Multimedia Appendix 1 [file games-v14-e81051-s001.pdf]

Supplementary Material

1. Development of the VR Intervention and Usability Study

Introduction

Prior to the clinical feasibility study, we conducted a usability study with healthy participants that served two main goals. Firstly, we wanted to determine the mean range of motion for healthy participants for the selected movement exercises. This later served as the ideal movement pattern in the most difficult condition, i.e., it was the movement pattern of the hologram overlaying the avatar of the user and the basis for the accuracy feedback after each exercise. Secondly, the usability study served as a test run to ensure that there were no unforeseen technical issues prior to the clinical study. Here, we will describe the usability study and highlight design choices in the implementation of the VR therapy within the methods section.

Methods

Participants. We recruited healthy individuals (*N* = 20) recruited via an online platform of the University of Würzburg prior to the study with the CLBP study participants. The sample had a mean age of 32.1 (*SD* = 15.2, 12 female participants). Exclusion criteria were chronic pain, mental or neurological disorders, pregnancy or intake of medication with effects on the central nervous system. Furthermore, no intake of alcohol, drugs or pain medication 12 h before the experiment (based on self-report) was allowed. If participants wore glasses, they had to be compatible with the HMD. Participants received 10 € for their participation.

The study protocol was approved by the Ethical Review Board of the Faculty of Medicine of the University of Würzburg (190/22-am). All participants signed informed consent before participation in accordance with the Declaration of Helsinki.

VR environment and UX design. The virtual body moved according to their real movements that were tracked via the handheld controllers and the Azure Kinect sensor. During the whole VR experience, participants wore a head-mounted display (HMD, Oculus Quest 2).

Embodiment: For the out-of-body illusion, a virtual mirror was placed in front of the avatar in order for the participants to observe the movements of their body. This procedure was previously demonstrated to induce full body illusions (e.g., [1,8]). To further enhance the impression that participants would own the virtual body, participants were asked to move their arms and legs in front of the mirror. As persons with chronic low back pain often hold maladaptive beliefs about their back and perceive it as vulnerable [3-5], we selected an avatar with a gender-neutral appearance that provides the impression of a flexible and indestructible rubber body.

Body Illusions: In a second step, a virtual scanning procedure took place and the avatar's position was moved such that subsequently participants observed it from a third person perspective while keeping full agency over it. With this, we wanted to induce an illusion similar to the one previously coined as "out of body illusion", where participants observed themselves from a third person perspective [1,7]. The distance from the third-person perspective should lead to a lesser connection with one's own body and its perception of pain, and thus to experience pain relief. This could be shown in a previous study investigating the effects of an "out-of-body illusion" on pain. For this, persons with chronic pain viewed themselves from a third person perspective: They wore a head-mounted display and watched a video stream of their own back, while it was being stroked with a stick. The participants experienced a full body illusion and a substantial pain reduction [9].

Gamification: We integrated several gamification elements, which had a positive impact in previous studies, to increase engagement motivation and fun ([12]; for an overview of possible elements, see [11]). The virtual environment was a cozy toy factory, in which participants had to teach toys their specific movement pattern. In the clinical study, there was also continuous performance feedback through the green hologram overlaying the avatar (this green overlay became darker, the closer the movements

matched the ones of the healthy participants). Additionally, a percentage score was provided visually after the movement exercises and also translated into auditory feedback that praised participants for their good performance.

Measures. Participants were asked to rate their VR experience (presence, satisfaction, user experience and gaming factor) on numerical rating scales (NRS) from 0 (*not at all*) to 10 (*extremely*). Furthermore, data of the following standardized questionnaires were collected: Simulator Sickness Questionnaire (SSQ; [6]), System Usability Scale (SUS; [2]) and items of the Embodiment Questionnaire (EQ; [10]). We furthermore adapted some items to assess the out-of-body illusion from [9].

Procedure. After arrival, participants provided informed consent and were given a detailed explanation of the experimental procedure. Next, participants answered socio-demographic questions. Then, the experimenter instructed the participants about the usage of the controller and equipped them with the HMD. Participants underwent the VR application including the out-of-body procedure and each movement exercise with 10 repetitions each. In total, the VR application lasted around 20 minutes. After finishing the application, participants filled in questionnaires.

Results

The usability study induced high levels of presence (M = 7.42, SD = 2.28) and was highly rated regarding usability on the SUS (M = 79.4, SD = 1.81). Regarding feasibility, there were only minor side effects following the SSQ (M = 6.23, SD = 2.84). Ratings regarding the avatar were low for humanness (M = 3.45, SD = 3.17) and realism (M = 2.97, SD = 3.00). We could find adequate embodiment levels regarding the avatar (EQ Agency: M = 3.94, SD = 1.29; EQ Ownership M = 1.01, SD = 1.46). However, we did not demonstrate a robust out-of-body illusion in terms of having the feeling to see the own body from the outside (all items $M \leq 3.7$ on a scale from 0-10). Based on the ROM values, we set the maximum range of motion for the clinical study.

Conclusion

The study yielded high levels of usability, and no side effects were revealed, hence we proceeded with the clinical feasibility study. For this, the ROM data of the healthy participants provided the ideal movement pattern for the CLBP study participants that the feedback accuracy was based upon. Maintaining an out-of-body illusion while moving in the virtual environment proved to be difficult (see other experimental setups, e.g., [7]), probably because of too strong kinesthetic bottom-up input from the movement, which led to cognitive conflicts. We consider the body illusion in the current VR intervention to be an out-of-virtual-body illusion.

References

[1] Bourdin P, Barberia I, Oliva R, Slater M. A virtual out-of-body experience reduces fear of death. PLoS One 2017;12(1):e0169343. doi:10.1371/journal.pone.0169343

[2] Brooke J. SUS-A quick and dirty usability scale. Usabil Evaluat Indus 1996;189(194):4-7.

[3] Darlow B. Beliefs about back pain: The confluence of client, clinician and community. Int J Osteopath Med 2016;20:53-61. doi:10.1016/j.ijosm.2016.01.005

[4] Darlow B, Dean S, Perry M, Mathieson F, Baxter GD, Dowell A. Easy to harm, hard to heal: Patient views about the back. Spine 2015;40(11):842-850. doi:10.1097/BRS.0000000000000901

[5] Darlow B, Perry M, Stanley J, Mathieson F, Melloh M, Baxter GD, Dowell A. Cross-sectional survey of attitudes and beliefs about back pain in New Zealand. BMJ Open 2014;4(5):e004725. doi:10.1136/bmjopen-2013-004725

[6] Kennedy RS, Lane NE, Berbaum KS, Lilienthal MG. Simulator Sickness Questionnaire: An enhanced method for quantifying simulator sickness. Int J Aviat Psychol 1993;3(3):203-220. doi:10.1207/s15327108ijap0303_3

[7] Lenggenhager B, Tadi T, Metzinger T, Blanke O. Video ergo sum: Manipulating bodily self-consciousness. Science 2007;317(5841):1096-1099. doi:10.1126/science.1143439

[8] Maselli A, Slater M. The building blocks of the full body ownership illusion. Front Hum Neurosci 2013;7. doi:10.3389/fnhum.2013.00083

[9] Pamment J, Aspell JE. Putting pain out of mind with an ‘out of body’ illusion. Eur J Pain 2017;21(2):334-342. doi:10.1002/ejp.927

[10] Peck TC, Gonzalez-Franco M. Avatar Embodiment. A Standardized Questionnaire. Front Virtual Real 2021;1. doi:10.3389/frvir.2020.575943
[11] Toda AM, Klock ACT, Oliveira W, Palomino PT, Rodrigues L, Shi L, Bittencourt I, Gasparini I, Isotani S, Cristea AI. Analysing gamification elements in educational environments using an existing Gamification taxonomy. Smart Learn Environ 2019;6(1):16. doi:10.1186/s40561-019-0106-1
[12] Winter C, Kern F, Gall D, Latoschik ME, Pauli P, Käthner I. Immersive virtual reality during gait rehabilitation increases walking speed and motivation: A usability evaluation with healthy participants and patients with multiple sclerosis and stroke. J NeuroEng Rehabil 2021;18(1):68. doi:10.1186/s12984-021-00848-w

2. Results

Table S1. Number of study participants indicating SSQ symptoms per session (mean ± SD).

SSQ symptom	Session 1 (8.73 ± 9.68)	Session 2 (7.27 ± 7.96)	Session 3 (7.06 ± 7.79)	Session 4 (6.03 ± 6.04)	Session 5 (4.99 ± 5.44)	Session 6 (4.36 ± 5.91)
General discomfort	2	0	0	0	0	0
Fatigue	0	1	0	0	1	0
Headache	0	0	0	0	0	0
Eye strain	5	4	4	3	3	2
Difficulty focusing	5	6	4	5	2	3
Increased salivation	0	0	0	0	0	0
Sweating	11	11	11	11	12	9
Nausea	0	0	0	1	0	0
Difficulty concentrating	2	0	1	0	0	0
Fullness of head	2	1	0	0	2	0
Blurred vision	3	2	2	0	0	2
Dizzy (eyes open)	0	0	0	0	0	0
Dizzy (eyes closed)	0	0	1	1	0	0

Vertigo	0	0	0	0	0	0
Stomach awareness	0	0	0	1	0	0
Burping	0	0	0	1	0	0

Note. SSQ range from 0 - 235.62.

Table S2. Frequency and means (± SD) over the course of intervention of most common activities listed in the PSFS. Note that lifting and carrying, walking and standing (shown in bold) improved clinically significant from pre to follow-up intervention (≥ 2 points, Heldmann et al., 2015).

Activity	*n*	Pre (*M* ± *SD*)	Post (*M* ± *SD*)	Follow-Up (*M* ± *SD*)
**Lifting (> 5 – 25 kg) & carrying (> 5 – 10 kg)**	8	3.88 ± 2.47	5.25 ± 2.19	7.00 ± 0.82
Bending down	7	5.29 ± 1.50	6.64 ± 2.46	6.00 ± 2.16
Climbing stairs	5	5.00 ± 0.82	5.75 ± 0.96	6.00 ± 1.41
**Walking**	4	4.25 ± 0.96	5.13 ± 2.46	7.00 ± 1.00
**Standing (> 30 min – 2h)**	4	4.00 ± 1.78	6.13 ± 1.25	6.25 ± 0.35
Sitting	4	6.00 ± 1.47	5.75 ± 2.06	5.67 ± 1.53

Note. Missing values at follow-up for lifting & carrying (*n* = 4), bending down (*n* = 3), climbing stairs (*n* = 2), walking (*n* = 1), standing (*n* = 2) and sitting (*n* = 1).

Table S3. Results of questionnaires measuring behavioral and psychological variables.

	Behavioral			
	Pre (*M* ± *SD*)	Post (*M* ± *SD*)	Follow-Up (*M* ± *SD*)	ANOVA
**BPS**	2.06 ± 2.15	1.56 ± 1.98	1.17 ± 1.62	$F(2, 34) = 4.53$, $p = .018$, $\eta^2_g = .036$<br>*pre to post:<br>$t(17) = 2.15$, $p = .046$, $d = .51$<br>*pre to follow-up:<br>$t(17) = 2.56$, $p = .019$, $d = .61$<br>*post to follow-up:

	Pre	Post	Follow-Up	ANOVA
				$t(17) = 1.28$, $p = .218$, $d = .30$
**PSFS**	4.67 ± 0.87	5.72 ± 1.7	6.22 ± 1.4	$F(2, 16) = 2.68$, $p = .099$, $\eta^2_g = .21$
**RDQ**	5.0 ± 3.11	5.86 ± 3.61	3.57 ± 3.01	$F(2, 26) = 4.73$, $p = .018$, $\eta^2_g = .08$<br>*pre to follow-up: $t(13) = 2.59$, $p = .022$, $d = .69$<br>*post to follow-up: $t(13) = 2.43$, $p = .030$, $d = .65$<br>*pre to post:<br>$t(13) = -1.21$, $p = .249$, $d = .32$
PGIC<br>*box*	//	3.21 ± 1.67	3.36 ± 1.86	$t(13) = -0.81$, $p = .435$, $d = .22$
PGIC<br>*slider*	//	5.0 ± 2.04	4.79 ± 1.37	$t(13) = 0.43$, $p = .671$, $d = .12$

Cognitive

	Pre<br>(*M* ± *SD*)	Post<br>(*M* ± *SD*)	Follow-Up<br>(*M* ± *SD*)	ANOVA
FABQ<br>*subscale physical activity*	8.64 ± 4.99	6.5 ± 4.57	8.29 ± 5.74	$F(2, 26) = 1.07$, $p = .358$, $\eta^2_g = .04$
FABQ<br>*subscale work*	8.29 ± 6.99	7.86 ± 10.64	7.64 ± 9.29	$F(2, 26) = 0.08$, $p = .92$, $\eta^2_g < .01$
TKS	20.29 ± 5.62	17.86 ± 4.7	18.36 ± 6.46	$F(2, 26) = 2.49$, $p = .103$, $\eta^2_g = .04$
PCS	14.29 ± 7.58	11.5 ± 8.79	11.71 ± 8.69	$F(2, 26) = 0.90$, $p = .42$, $\eta^2_g = .02$
		Psychological		
PROMIS 29<br>*subscale physical function*	4.52 ± 0.43	4.38 ± 0.64	4.5 ± 0.6	$F(2, 26) = 0.71$, $p = .503$, $\eta^2_g = .01$

PROMIS 29 *subscale anxiety*	1.32 ± 0.36	1.38 ± 0.4	1.3 ± 0.26	$F(1.37, 17.86) = 0.18$, $p = .759$, $\eta^2_g = .01$
PROMIS 29 *subscale depressive symptoms*	1.25 ± 0.4	1.39 ± 0.41	1.39 ± 0.36	$F(1.24, 16.14) = 1.07$, $p = .333$, $\eta^2_g = .03$
PROMIS 29 *subscale fatigue*	2.5 ± 0.55	2.59 ± 0.69	2.61 ± 0.86	$F(2, 26) = 0.24$, $p = .787$, $\eta^2_g = .01$
PROMIS 29 *subscale sleep disturbance*	3.11 ± 0.59	2.88 ± 0.66	2.98 ± 0.74	$F(2, 26) = 1.02$, $p = .376$, $\eta^2_g = .02$
PROMIS 29 *subscale ability to participate in social roles and activities*	1.89 ± 0.59	1.88 ± 0.75	2.07 ± 0.64	$F(2, 26) = 0.96$, $p = .395$, $\eta^2_g = .02$
PROMIS 29 *subscale pain interference*	2.29 ± 0.76	2.29 ± 0.85	1.98 ± 0.74	$F(2, 26) = 1.68$, $p = .205$, $\eta^2_g = .03$

Note. Questionnaires yielding significant and marginal significant differences are printed in bold. BPS = Back Performance Scale (range from 0-15), FABQ = Fear Avoidance Belief Questionnaire (range from 0-24 subscale physical activity, 0-42 subscale work), PROMIS 29 = Patient-Reported Outcomes Measurement Information System (range from 1-5, 0-10 for NRS pain), PCS = Pain Catastrophizing Scale (range from 0-52), PGIC = Patient Global Impression of Change (range from 1-7 box, 0-10 slider), PSFS = Patient-Specific Functional Scale (range from 0-10, scores of activities were averaged), RDQ = Roland-Morris Disability Questionnaire (range from 0-24), TKS = Tampa Scale of Kinesiophobia (range from 11-44).

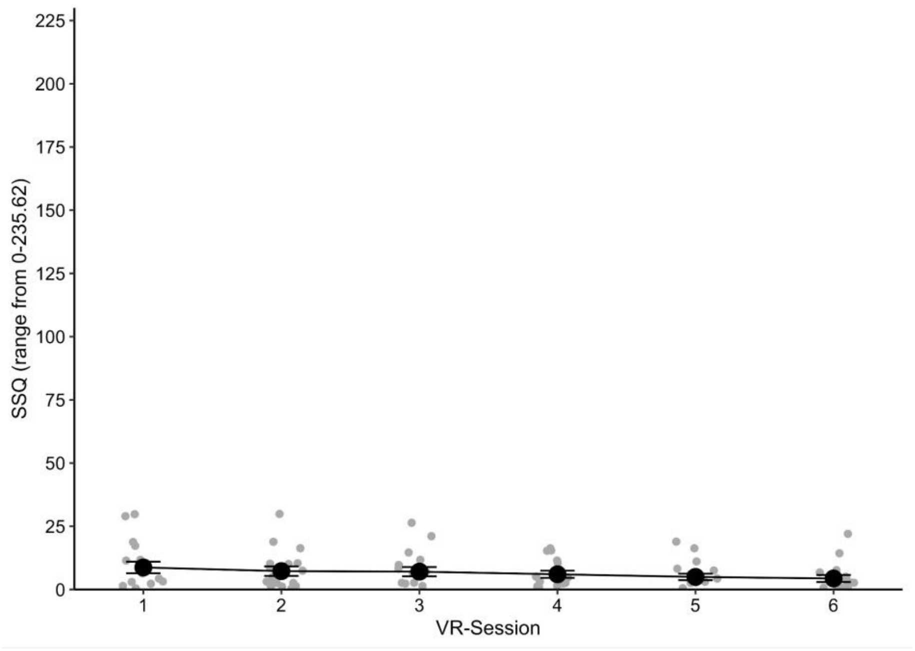

Fig. S1. Mean ± SE of SSQ scores per each VR session.

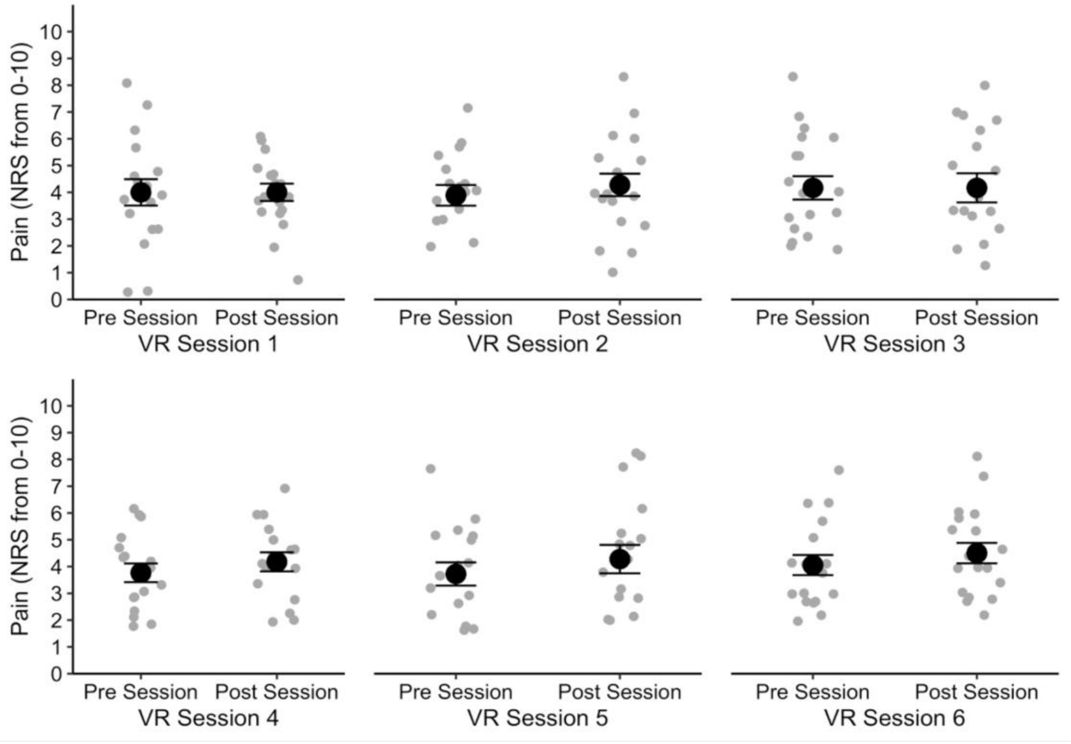

Fig. S2. Mean ± SE of pain ratings pre and post each VR session.
