# Supplementary material for: A Novel Virtual Reality Intervention Combining Movement Exercises and Body Illusions for the Treatment of Chronic Back Pain: Prospective Feasibility Study
Source: JMIR Serious Games. 2026 Mar 30;14:e81051. doi: 10.2196/81051 (PMC13035036; doi:10.2196/81051)
Supplement: Multimedia Appendix 2 [file games-v14-e81051-s002.pdf]

Supplementary Material 2

Little's MCARTest

Table S1. Results of Little's MCAR Test per outcome variable.

**Outcome variable**	$n_{missing}$	χ^2	**df**	***p***
Pain rating (pain diary)	Pre: 0<br>Post: 1<br>Follow-Up: NA	0.19	1	.660
ROM	Pre: 0<br>Post: 0<br>Follow-Up: 0	NA	NA	NA
BPS	Pre: 0<br>Post: 0<br>Follow-Up: 0	NA	NA	NA
PSFS	Pre: 0<br>Post: 0<br>Follow-Up: 8	0.87	2	.649
RDMQ	Pre: 0<br>Post: 1<br>Follow-Up: 3	1.44	4	.837
PGIC box	Post: 1<br>Follow-Up: 3	4.79	2	.091
PGIC slider	Post: 1<br>Follow-Up: 3	4.48	2	.106
FABQ Akt	Pre: 0<br>Post: 1<br>Follow-Up: 3	4.90	4	.298
FABQ Work	Pre: 0<br>Post: 1<br>Follow-Up: 3	2.24	4	.691
TAMPA	Pre: 0<br>Post: 0<br>Follow-Up: 1	1.70	2	.427
PCS	Pre: 0<br>Post: 1<br>Follow-Up: 3	7.03	4	.135
PROMIS 29<br>*subscale physical function*	Pre: 0<br>Post: 1<br>Follow-Up: 3	2.98	4	.561
PROMIS 29<br>*subscale anxiety*	Pre: 0<br>Post: 1<br>Follow-Up: 3	4.50	4	.343
PROMIS 29<br>*subscale depressive symptoms*	Pre: 0<br>Post: 1<br>Follow-Up: 3	8.68	4	.069
PROMIS 29<br>*subscale fatigue*	Pre: 0<br>Post: 1<br>Follow-Up: 3	5.58	4	.233
PROMIS 29<br>*subscale sleep disturbance*	Pre: 0<br>Post: 1<br>Follow-Up: 3	1.21	4	.877
PROMIS 29<br>*subscale ability to participate in social roles and activities*	Pre: 0<br>Post: 1<br>Follow-Up: 3	1.45	4	.835
PROMIS 29<br>*subscale pain interference*	Pre: 0<br>Post: 1<br>Follow-Up: 3	3.62	4	.461
